# Cytokine Networks in Triple-Negative Breast Cancer: Mechanisms, Therapeutic Targets, and Emerging Strategies

**DOI:** 10.3390/biomedicines13081945

**Published:** 2025-08-08

**Authors:** María Rosado-Sanz, Nuria Martínez-Alarcón, Adrián Abellán-Soriano, Raúl Golfe, Eva M. Trinidad, Jaime Font de Mora

**Affiliations:** Laboratory of Cellular and Molecular Biology and Clinical and Translational Research in Cancer, Health Research Institute Hospital La Fe, Avenida Fernando Abril Martorell 106, 46026 Valencia, Spain

**Keywords:** triple-negative breast cancer, cytokines, therapeutic targets, immunomodulation, clinical trial

## Abstract

Triple-negative breast cancer (TNBC) remains a challenging subtype of breast cancer due to its aggressive nature and lack of targeted therapies. Cytokines play a pivotal role in shaping the tumor microenvironment, modulating tumor progression, immune evasion, and therapy resistance. In this review, we discuss the complex cytokine networks involved in TNBC biology, highlighting their contribution to key oncogenic processes, including proliferation, angiogenesis, epithelial–mesenchymal transition, and immunomodulation. We also summarize current and emerging cytokine-targeted therapeutic strategies, including monoclonal antibodies, bispecific antibodies, cell-based therapies, and cytokine-armed CAR-T and CAR-NK cell approaches, with a focus on clinical implications and future directions.

## 1. Introduction

Breast cancer (BC) is the most frequently diagnosed tumor among women worldwide. It comprises a biologically and clinically heterogeneous group of tumors, classified into subtypes based on hormone receptor and human epidermal growth factor receptor 2 (HER2) expression status. Among these, triple-negative breast cancer (TNBC), defined by the absence of progesterone and estrogen receptors (ERs) and the lack of HER2 overexpression or gene amplification, accounts for approximately 10–15% of all new diagnoses and is associated with the poorest prognosis [1].

TNBC exhibits an aggressive clinical course, characterized by rapid progression, early recurrence, and distinct metastatic patterns. The disease carries a particularly poor outcome, with a median overall survival of approximately 10.2 months in the metastatic setting. Five-year survival rates are markedly reduced compared to other subtypes, with outcomes of 65% in patients with regional disease and only 11% when distant metastases are present [2,3].

Despite being biologically diverse, resulting in variable clinical presentations and epidemiological patterns, TNBC remains unique among BCs due to the absence of approved tumor-specific targeted therapies. Chemotherapy remains the cornerstone of systemic treatment in TNBC. Standard regimens typically incorporate taxanes and anthracyclines, while platinum-based agents are frequently employed, particularly in the neoadjuvant and metastatic settings, due to their efficacy in DNA damage response–deficient tumors. Resistance to chemotherapy constitutes a major barrier to effective cancer treatment, particularly in the metastatic context, where it is responsible for approximately 90% of therapeutic failures [4,5]. Ongoing research efforts are essential to identify actionable molecular targets and overcome mechanisms of immune resistance [6].

In this context, cytokines have emerged as relevant players, not only as potential biomarkers for diagnosis, prognosis, and treatment response but also as active modulators of tumor behavior. These low-molecular-weight proteins (6 to 70 kDa) function as key signaling molecules within the tumor microenvironment (TME), orchestrating the dynamic interactions between cancer cells, stromal components, and immune infiltrates [7]. In TNBC, where the TME is typically characterized by chronic inflammation, immune evasion, and stromal remodeling, cytokines contribute to the establishment of a pro-tumorigenic niche that favors disease advancement [8,9].

Cytokines regulate a wide array of cellular functions, including differentiation, proliferation, apoptosis, and survival, and are involved in nearly all stages of cancer development. Within the TME, they influence several hallmarks of cancer by promoting tumor cell growth, stimulating angiogenesis to sustain tumor vascularization, supporting metastatic potential through modulation of cellular plasticity and motility, and contributing to tumor-promoting inflammation by modulating immune cell recruitment and function [7,10,11].

**Figure 1:**
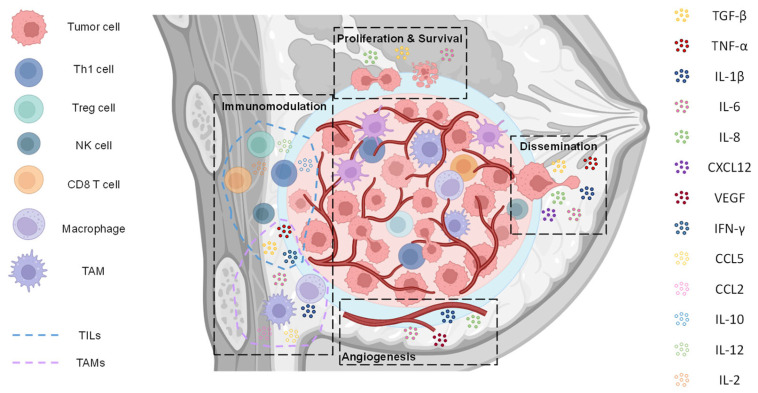
Summary of main cytokines implicated in the development of TNBC. Cytokines are listed on the right side, while the different types of cells are on the left.

Given their multifaceted roles in cancer biology and immune regulation, cytokines represent both a biological challenge and a therapeutic opportunity. In this review, we examine the most relevant cytokines implicated in TNBC (Figure 1) and explore their potential as targets for therapy, focusing particularly on those currently being investigated in clinical trials. We highlight how cytokine-based strategies could contribute to the development of more personalized and effective treatments for patients affected by this challenging BC subtype.

## 2. Tumor Proliferation and Survival

Several uncontrolled intracellular signaling pathways supporting unlimited growth and apoptosis resistance are central to the development of TNBC. Among these, the phosphatidylinositol-3-kinase (PI3K)/protein kinase B (Akt)/mammalian target of rapamycin (mTOR) pathway is particularly important, as it is frequently activated by mutations in *PIK3CA*, *AKT1*, or loss of phosphatase and tensin homolog (PTEN). These alterations occur in about 25% of TNBC patients. Activating this pathway inhibits pro-apoptotic processes and advances cell cycle progression, therefore promoting chemoresistance and aggressive tumor behavior [12,13,14]. Another major driver of TNBC growth is the mitogen-activated protein kinase (MAPK)/extracellular signal-regulated kinase (ERK) pathway (RAS/RAF/MEK/ERK cascade). Upstream signals, such as epidermal growth factor receptor (EGFR) overexpression, a feature frequently observed in basal-like TNBCs, commonly activate this pathway. EGFR activation triggers both PI3K/Akt and MAPK signaling, promoting downstream processes that drive proliferation, migration, and angiogenesis [15,16,17].

In TNBC, the Janus kinases (JAK)/signal transducer and activator of transcription 3 (STAT3) pathway is often activated to regulate gene expression involved in proliferation, survival, angiogenesis, and immune evasion. Cytokines such as interleukin-6 (IL-6), which are profusely produced by TNBC cells as well as the neighboring TME, often promote this pathway. High IL-6 levels are linked to unfavorable clinical outcomes and therapeutic resistance [17,18]. Inhibiting IL-6/JAK/STAT3 signaling has been found to slow tumor development and reinstate chemosensitivity [17,18]. By molding the TME and strengthening autocrine survival loops, cytokines are crucial in TNBC development. Along with IL-6, interleukin-8 (IL-8) is often overexpressed in TNBC and serves as a strong promoter of cell proliferation, angiogenesis, and stemness [19]. For its part, TGF-β contributes to tumor proliferation by regulating CSCs [20] and modulates apoptotic signals [21]. Below, these important cytokines in TNBC tumor proliferation and survival are described.

### 2.1. IL-6

TNBC development depends critically on IL-6, a pleiotropic cytokine implicated in inflammatory responses and hematopoiesis. The production of IL-6 by immunological and stromal cells acts in a paracrine manner to drive neovascularization and inflammation-driven carcinogenesis, thereby enabling autonomous TNBC cell proliferation [22]. Its activity is controlled by binding to the IL-6 receptor (IL-6R), which might be membrane-bound or soluble. The soluble form of IL-6R (sIL-6R), which consists of the extracellular domain of the receptor, binds IL-6 with an affinity comparable to that of the membrane-bound IL-6R. The resulting IL-6/sIL-6R complex can activate intracellular signaling cascades, primarily via the JAK/STAT3 and MAPK pathways, in cells that lack membrane-bound IL-6R. This mechanism is referred to as IL-6 trans-signaling [23].

IL-6 binding to its receptor leads to dimerization of not only the signal-transducing subunit gp130, which activates primarily JAK1, but also JAK2 and TYK2 in certain contexts. Activated JAK1 phosphorylates STAT3, which then dimerizes, translocates to the nucleus, and binds DNA to regulate the expression of genes involved in cell proliferation [24]. IL-6 acts on tumor cells to induce STAT3 target genes that promote proliferation and survival, creating a feedforward autocrine loop [25]. One important target of proliferation is the *CCND1* gene, which encodes a protein crucial for cell cycle progression from the G1 to the S phase. Overexpression of cyclin D1 accelerates the cell cycle, promoting uncontrolled proliferation [26]. Additionally, IL-6/STAT3 signaling induces the expression of the *MYC* oncogene. Elevated c-Myc levels increase the transcription of genes driving stemness, metabolism, and proliferation [18]. STAT3 also induces factors that promote angiogenesis (e.g., vascular endothelial growth factor (VEGF), see Section 4), invasiveness/metastasis (e.g., metalloproteinases (MMPs), see Section 3), and immunosuppression (e.g., IL-10, TGF-β, see Section 5) [27,28]. Other transcriptional targets of survival in the IL-6/STAT3 pathway are *BCL2* (B-cell leukemia/lymphoma 2) and *MCL-1* (myeloid cell leukemia-1) genes [29]. Their coding proteins prevent the release of pro-apoptotic molecules from the mitochondrial intermembrane space, hence halting caspase activation and inhibiting programmed cell death [29]. The IL-6/STAT3 signaling pathway is often constitutively triggered in TNBC, hence promoting tumor development and dismal prognosis [29]. This constant activation aids the survival and self-renewal of cancer stem-like cells (CSCs), abundant in TNBC and linked to resistance to standard chemotherapies.

Additionally, IL-6 signaling interacts with other pro-proliferative pathways, such as PI3K/Akt and MAPK/ERK, therefore enhancing their effects and providing a cellular milieu favorable for tumor growth and survival [30]. IL-6 also activates the PI3K/Akt signaling pathway at the same time, hence promoting cell survival by phosphorylating and deactivating pro-apoptotic proteins like Bcl2-associated agonist of cell death and by triggering pro-survival transcription factors, including Nuclear Factor Kappa B (NF-κB) [31].

IL-6 secreted by cells in the TME, like cancer-associated fibroblasts (CAFs), tumor-associated macrophages (TAMs), and tumor cells, creates a pro-survival niche that supports TNBC development [18]. Furthermore, IL-6 signaling has been proven to improve the survival of breast cancer stem-like cells (BCSCs), a subpopulation rich in TNBC naturally resistant to chemotherapy and perhaps also capable of avoiding immune responses [32]. High IL-6 levels in TNBC correlate with worse prognosis, emphasizing its relevance as a possible therapeutic target [33].

Given the central role of IL-6 in TNBC, several clinical trials have explored strategies to inhibit its signaling (Table 1). One such approach involves tocilizumab, a humanized monoclonal antibody (mAb) that blocks both soluble and membrane-bound IL-6 receptors, thereby inhibiting IL-6-driven signaling pathways implicated in tumor progression, particularly in TNBC. Beyond its immunomodulatory role, tocilizumab demonstrates direct anti-tumor effects by inhibiting epithelial–mesenchymal transition (EMT), cancer cell stemness, and stromal cell activation, processes key to TNBC metastasis and resistance. Mesenchymal stem-like tumors, a subtype of TNBC characterized by poor response to chemotherapy, exhibit MAPK pathway activation and an IL-6-driven inflammatory cascade. Combining tocilizumab with chemotherapy delays progression in preclinical models compared to chemotherapy alone, suggesting that IL-6 targeting may enhance treatment efficacy in this subset [34]. A Phase II trial (NCT05846789) is currently evaluating the addition of tocilizumab to standard-of-care (SOC) chemotherapy in Black and non-Black patients with metastatic TNBC (mTNBC) or ER-low BC to assess racial differences [35].

Several clinical trials are evaluating ruxolitinib, a JAK1/2 inhibitor that targets the IL-6/JAK2/STAT3 axis. By disrupting this pathway, ruxolitinib may reduce tumor progression, immune suppression, and cytokine-driven tumor-immune interactions, thus enhancing sensitivity to other therapies. Multiple clinical trials are exploring its potential in TNBC (Table 1). NCT06008275 is evaluating ruxolitinib combined with neratinib, an EGFR inhibitor, to target synergistic signaling pathways in mTNBC. NCT02876302, a Phase II trial, assesses ruxolitinib with neoadjuvant chemotherapy in inflammatory BC (IBC), focusing on pathway inhibition and enhanced chemotherapy response. NCT02041429 determined a recommended Phase II dose of 15 mg twice a day of ruxolitinib with weekly paclitaxel, showing manageable toxicity and signs of clinical benefit, including stable disease in 63% of patients and a durable response in one IBC case. Additionally, NCT03012230 is a Phase I trial testing ruxolitinib in combination with the anti-PD-1 immunotherapy pembrolizumab in advanced/mTNBC.

### 2.2. IL-8

Along with IL-6, IL-8 is consistently overexpressed in TNBC and has emerged as a critical promoter of tumor progression [36,37], proliferation [38,39], EMT, migration, invasion (see Section 3) [40], and angiogenesis (see Section 4) [41].

IL-8 exerts its effects primarily through the binding to C-X-C motif chemokine receptors 1 and 2 (CXCR1 and CXCR2), activating downstream MAPK/ERK, PI3K/Akt, and NF-κB pathways, all of which are commonly deregulated in TNBC [42]. Upon binding to its receptors CXCR1 and CXCR2, IL-8 activates G-protein-coupled signaling cascades that lead to the activation of key intracellular kinases, including PI3K, ERK1/2, and p38 MAPK [43]. As in IL-6 signaling, IL-8 engagement of PI3K results in the inhibition of apoptosis and metabolism control [44]. Also, ERK1/2 activation promotes cell cycle progression. Moreover, IL-8-driven activation of NF-κB amplifies transcription of additional pro-survival and pro-inflammatory genes, establishing a feedback loop that sustains tumor-promoting inflammation and enhances TNBC cell fitness [45]. These pathways drive cell cycle progression, inhibit apoptosis, and promote survival under stress conditions such as hypoxia or chemotherapy exposure [36,46].

Beyond its mitogenic functions, IL-8 plays a central role in maintaining the population of BCSCs, which are known to be enriched in TNBC and strongly linked to chemoresistance and tumor relapse. IL-8 signaling supports the self-renewal and survival of CSCs through upregulation of *ALDH1A3*, *BCL2*, and EMT-related genes like *ZEB1* and *TWIST1* [40]. In TNBC, IL-8 also recruits myeloid-derived suppressor cells (MDSCs), supporting stemness [47], and upregulates breast cancer resistance proteins, contributing to doxorubicin resistance [48].

High serum IL-8 has been correlated with BC progression [49], so inhibiting IL-8 signaling is postulated as a promising strategy. Reparixin (CXCR1/2 inhibitor) reduced ALDH^+^ and CD24^−^/CD44^+^ CSCs and metastases in preclinical models [50]. Reparixin has been evaluated in two BC clinical trials (NCT02001974 and NCT02370238), concluding that its administration is safe and that tumor progression increases in the arm with high doses of reparixin (Table 1).

### 2.3. TGF-β

Transforming Growth Factor-beta (TGF-β) is a multifunctional family of cytokines that exert a pleiotropic effect in TNBC [51,52]. TGF-β promotes tumor progression by inducing EMT, enhancing metastasis, and modulating the TME to favor immune evasion (see Section 3 and Section 5). TGF-β also plays a key role in linking tumor cell proliferation and therapy resistance by regulating CSCs [20]. In TNBC patients treated with chemotherapy, elevated TGF-β signaling was found together with markers of both mesenchymal and epithelial CSCs [53]. Chemotherapy agents such as paclitaxel can induce TGF-β expression, which in turn promotes CSC proliferation and enhances tumorigenic potential [54]. Similarly, established epirubicin-resistant TNBC cells display increased stemness, suggesting that TGF-β contributes to therapeutic resistance and tumor regrowth by supporting the CSC population [55,56].

Beyond its effects on stemness, TGF-β also influences cell survival by modulating pro- and anti-apoptotic factors [57]. In epirubicin-resistant TNBC cells, this is reflected by elevated levels of the anti-apoptotic protein Bcl-2, supporting the idea that resistance involves evasion of apoptosis. The PI3K/Akt pathway further complicates this scenario, as it promotes survival and can interact with TGF-β signaling. Specifically, Akt can bind and sequester Smad3, blocking TGF-β-driven pro-apoptotic signals and shifting the balance toward survival [54]. This highlights a dynamic interplay where TGF-β and PI3K/Akt signaling cooperate in resistant TNBC cells to favor stemness and survival over cell death.

Given the central role of TGF-β in driving therapeutic resistance, survival, and stemness pathways in TNBC, efforts to target this axis have attracted considerable interest as a strategy to limit tumor progression and overcome drug resistance. Various anti-TGF-β strategies are under clinical and preclinical investigation, including mAbs, small-molecule receptor inhibitors, and ligand traps that block receptor binding [58]. The related clinical trials are described in the next section (Table 1).

## 3. Dissemination: EMT, Invasion, Migration, and Metastasis

EMT, invasion, migration, and metastasis are key processes driving tumor dissemination. During EMT, epithelial tumor cells acquire a mesenchymal phenotype, characterized by fibroblast-like morphology, loss of cell–cell adhesion, and increased motility and invasiveness [59]. While EMT and the reverse process, mesenchymal-epithelial transition (MET), are physiological during development (type I EMT) and tissue repair (type II EMT), cancer cells (type III EMT) exploit these programs to enhance their metastatic potential [60]. Notably, tumor cells may display mesenchymal traits without fully losing epithelial features [61].

Cytokines such as tumor necrosis factor alpha (TNF-α) and TGF-β, secreted by TAMs and other components of the TME, play a pivotal role in inducing EMT in TNBC, promoting migration and invasion [62]. This involves activation of pathways like NF-κB and MAPK, upregulation of EMT transcription factors (e.g., ZEB, TWIST, and SNAIL), and downregulation of epithelial markers like E-cadherin [63]. EMT is also linked to the generation of BCSCs, which drive tumor growth, metastasis, therapy resistance, and relapse [64]. Additional cytokines and chemokines, including IL-1β, IL-6, IL-8, and C-X-C motif chemokine ligand 12 (CXCL12), further support TNBC dissemination and are under investigation as therapeutic targets.

### 3.1. TGF-β

TGF-β is essential for normal mammary gland development but also plays a critical role in BC progression. Binding of TGF-β isoforms to their type II receptor triggers complex formation with the type I receptor, phosphorylation of suppressor of mother against decapentaplegic 2/3 (SMAD2/3), and assembly of SMAD2/3/4 complexes that translocate to the nucleus to regulate gene expression [65,66,67]. TGF-β also activates noncanonical pathways, including NF-κB, Akt, and ERK [58].

In early breast tumorigenesis, TGF-β exerts tumor-suppressive effects through growth inhibition [40,68]. However, as cancer advances, cells become resistant to these effects, and TGF-β switches to a tumor-promoting role, enhancing EMT, invasiveness, metastatic potential, and resistance to therapy [69,70,71,72,73,74]. Moreover, TGF-β contributes to immune evasion by impairing tumor-infiltrating lymphocyte (TIL) activity, altering macrophage polarization, recruiting and promoting differentiation of regulatory T cells (Tregs), and suppressing dendritic cell (DC) function [75,76,77] (see Section 5). Elevated TGF-β levels have been associated with poor responses to anti-programmed death protein 1 (anti-PD-1)/anti-programmed death-ligand protein 1 (PD-L1) therapies in TNBC [75].

These insights have prompted clinical strategies combining TGF-β blockade with immune checkpoint inhibition (Table 1). For example, bispecific antibodies targeting TGF-β and PD-L1 (e.g., BiTP) are under development [78] (see Section 6). Similarly, Vigil (gemogenovatucel-T), a triple-acting vaccine that reduces TGF-β1/β2 and furin, combined with durvalumab, showed encouraging preliminary efficacy in advanced TNBC (NCT02725489) [79]. Other approaches include fresolimumab (anti-TGF-β mAb, all isoforms, NCT01401062) and NIS793 (anti-TGF-β1 mAb, NCT02947165), with the latter combined with spartalizumab showing preliminary activity in advanced solid tumors [78]. A Phase I study (NCT02672475) was recently completed, assessing galunisertib in combination with chemotherapy in metastatic AR-negative TNBC with the primary objective of establishing the maximum tolerated dose, but the results are pending publication. While these early-phase studies primarily focused on safety, they set the stage for future trials to explore whether disrupting TGF-β signaling can meaningfully enhance chemotherapy efficacy, particularly in resistant disease settings where conventional treatments fail. Further clinical validation will be critical to determine whether TGF-β inhibitors can fulfill this therapeutic promise and how best to integrate them into combination strategies targeting multiple resistance mechanisms.

### 3.2. TNF-α

TNF-α is a key pro-inflammatory cytokine in the TME of BCs, produced by stromal cells, particularly M1-polarized TAMs, and by cancer cells themselves. TNF-α, a type II transmembrane protein of the TNF/TNFR superfamily, exerts its biological effects both as a membrane-bound and soluble molecule by interacting with two receptors: TNFR1 (TNFRSF1A), broadly expressed on many cell types, including epithelial tumor cells, and TNFR2 (TNFRSF1B), predominantly found on hematopoietic cells and in BC tissues [80,81]. TNF-α exhibits a dual role in BC, inducing apoptosis in some contexts [82], but more often activating pro-survival and pro-tumorigenic pathways [83,84]. Its signaling through TNFR1 or TNFR2 stimulates pathways such as MAPK/ERK, PI3K/Akt, p38/MAPK, JNK, STAT3, and NF-κB, ultimately promoting proliferation, survival, and inflammation [82,84]. The outcome of TNF-α signaling depends on the tumor’s molecular profile and microenvironmental cues [85,86,87].

A major contribution of TNF-α to BC progression is the induction of EMT and cancer stemness. TNF-α exposure transforms both healthy breast epithelial cells and BC-derived cell lines, including ER^+^ (e.g., MCF7 and T47D) and TNBC (e.g., BT-549 and Hs578T), into cells with fibroblast-like morphology, reduced cell–cell adhesion, and cytoskeletal reorganization [64,88,89,90,91]. This is accompanied by downregulation of epithelial markers (E-cadherin and β-catenin) and upregulation of mesenchymal markers (vimentin, N-cadherin, and fibronectin), as well as expansion of the cluster of differentiation 24^−^ (CD24^−^)/CD44^+^ cancer stem cell-like population [88,90,92,93]. In basal-like BC cells, TNF-α can further drive Notch pathway activation via NF-κB-dependent Jagged1 induction, enhancing BCSCs expansion [92,93,94]. When combined with TGF-β, TNF-α promotes transition toward more aggressive, claudin-low phenotypes with heightened drug resistance [88].

TNF-α also promotes migration and invasion by upregulating EMT-associated transcription factors (e.g., SNAIL, TWIST, and ZEB families) and metastasis-related proteases (e.g., matrix metalloproteinase-9 (MMP9) through NF-κB, STAT3, and c-Jun activation [95]. Importantly, TNF-α contributes to angiogenesis (see Section 4), further supporting tumor dissemination.

Despite its early promise as an anti-cancer agent, systemic administration of TNF-α in clinical trials led to unacceptable toxicity while showing limited efficacy [96]. Given TNF-α’s context-dependent effects, being pro-apoptotic at high doses but pro-tumorigenic when chronically produced within the TME, therapeutic strategies have shifted toward its neutralization. Preclinical studies with TNF-α inhibitors, including infliximab and antibodies targeting transmembrane TNF-α, have demonstrated reduced tumor growth, metastasis, and synergistic effects with chemotherapy in BC models [97]. However, clinical data remain scarce. A small study with etanercept in metastatic BC (mBC) showed it was well tolerated and reduced systemic inflammation (C-C motif chemokine ligand 2 (CCL2), IL-6 levels) but without meaningful clinical responses [98]. These findings highlight the complexity of targeting TNF-α in BC: while blocking its chronic signaling may offer therapeutic benefit (see Section 5), further clinical validation is required.

### 3.3. IL-1β

Interleukin-1 (IL-1) plays a pivotal role in both physiological and pathological states, including angiogenesis, tumor growth, and metastases [99]. IL-1 can be directly produced by cancer cells or can educate the cells of the TME to produce IL-1 [100], illustrating the complex signaling and interplay between the tumor and surrounding cells. IL-1α is localized in the cytosol and acts within the intracellular environment, whereas IL-1β is secreted extracellularly and may act on surrounding tissues [101]. Once IL-1β binds to its receptor IL-1R1, downstream signaling leads to the activation of NF-κB-dependent genes, which increases the migratory activity of BC cells and upregulates other cytokines like IL-6 or IL-8 under oxygen deprivation [102,103,104].

Studies have found that tumor cell IL-1β drives tumor progression in TNBC [105], invasion, metastases [106], stemness, and EMT [107]. IL-1β also contributes to angiogenesis (see Section 4), TAM recruitment, and immunosuppression (see Section 5). The association between IL-1β expression and metastatic potential for BC has been demonstrated both in vitro [108] and in vivo [109]. In mouse models, blocking IL-1R with agents like Anakinra (a recombinant polypeptide antagonist of IL-1R) reduced the development and progression of bone metastases from BC [109]. Similarly, IL-1β-deficient mice injected orthotopically with BC cells showed initial tumor growth, followed by regression linked to recruitment of alternative inflammatory monocytes and increased IL-12 secretion, promoting anti-tumor immunity and CD8^+^ T-cell activation [110]. Furthermore, combining PD1/PD-L1 inhibition with IL-1β inhibition produced synergistic effects: in preclinical studies, anti-IL-1β antibodies combined with PD-1 blockade completely halted tumor progression [110].

Several IL-1β inhibitors are FDA-approved for rheumatological and autoimmune conditions: Anakinra, Canakinumab (mAb IL-1β), and Rilonacept (a dimeric fusion protein combining two IL-1 receptors with an Fc immunoglobulin tail). IL-1β inhibition is being explored in solid tumors, including BC and TNBC (NCT02900664, NCT01802970, NCT03742349, and NCT06710197) (Table 1). A clinical trial proposed evaluating novel immunotherapy combinations (including IL-1β blockade) in locally advanced or mTNBC was terminated due to sponsor decisions rather than safety concerns (NCT03742349). Another ongoing clinical trial (NCT06710197) is assessing changes in the TME after 14 days of preoperative IL-1-inhibiting therapy (Anakinra) in patients with early BC (including TNBC and ER-low positive tumors). Collectively, these findings support IL-1β inhibition as a promising strategy for TNBC, although further investigation is needed.

### 3.4. IL-6

As mentioned in the previous section, IL-6 impacts cancer progression via key signaling cascades, notably the IL-6/JAK/STAT3 pathway, which plays a critical role in malignant tumor development, as well as invasion and metastasis [24]. IL-6 promotes EMT [111] and is elevated in chronic inflammatory conditions and many cancers [112]. In cancer, high IL-6 levels stimulate hyperactivation of JAK/STAT3, often linked to poor BC patient outcomes [113]. IL-6 is produced by multiple TME cell types, including tumor-infiltrating immune cells, stromal cells, and tumor cells [27,112,114]. Exogenous IL-6 promotes BC metastasis [114] and EMT [115]. Several IL-6 blocking strategies are under review in multiple trials combined with chemotherapy (Table 1, see Section 2).

### 3.5. IL-8

IL-8 (also known as CXCL8) is a pro-inflammatory chemokine produced by macrophages, endothelial cells, and epithelial cells in response to infection or injury, inducing granulocyte chemotaxis, phagocytosis, and oxidative burst [116]. Tumor-derived IL-8 alters TME immune composition, promoting immunosuppression (via neutrophil and MDSC infiltration) and enhancing angiogenesis and invasion [117]. IL-8 is a potent inducer of EMT and adhesion properties and has gained migratory and invasive capabilities through PI3K/Akt pathways [40]. Alternative signaling pathways, such as phospholipase C (PLC), protein kinase C, focal adhesion kinase (FAK), and Rho-family GTPases, are also activated by IL-8 after binding with CXCR1 and CXCR2, promoting the migration of cancer cells [36,40,118,119,120]. In Table 1, promising strategies to block IL-8 signaling are detailed.

### 3.6. CXCL12

CXCL12, also known as stromal cell-derived factor-1 (SDF-1), plays a critical role in leukocyte trafficking, tissue regeneration [121,122], bone marrow stem cell homing [123], cardiovascular development [124], and neuronal guidance [125]. Seven CXCL12 isoforms have been identified, with α and β being the most studied [126]. CXCL12-α is the most abundant subtype; CXCL12-β shows stronger pro-angiogenic properties and is enriched in hypervascular organs, such as the kidney, liver, and spleen [126], while CXCL12-γ, predominantly found in low-vascularization tissues like the heart and brain [127], has the highest affinity for CXCR4 and the longest downstream activity [128]. CXCL12-γ expression in primary tumors has been linked to BC metastasis [129].

CXCL12 signals mainly through two receptors: CXCR4 and CXCR7 [130]. CXCR4 is a G-protein-coupled receptor (GPCR) expressed on lymphocytes, endothelial cells, hematopoietic stem cells, stromal fibroblasts, and cancer cells [131]. CXCL12 binding to CXCR4 activates G-protein signaling (via Gα and Gβγ subunits), leading to downstream cascades that promote gene transcription, calcium influx, chemotaxis, survival, and proliferation [132] through Ras/MAPK [133], PI3K [134], and PLC [135] signaling. CXCR4 can also activate alternative pathways [136,137,138]. In contrast, CXCR7 signals predominantly via β-arrestin rather than G-proteins [139], functioning as a high-affinity scavenger receptor that limits CXCL12 availability and dampens CXCR4 activity [140,141].

The CXCL12-CXCR4 axis promotes invasion and metastasis in many cancers [142,143,144,145], including TNBC. Various approaches have targeted this axis. Saikosaponin A, a bioactive compound from *Radix bupleuri*, reduced TNBC growth and metastasis by downregulating CXCR4 and interfering with PI3K/Akt/mTOR and MMP pathways [146]. Dipeptidyl peptidase 4 inhibitors highlighted the importance of CXCL12/CXCR4/mTOR in EMT, although their pro-metastatic effects could be mitigated by metformin [147]. The CXCL12-CXCR7 axis also contributes to metastasis; in TNBC models, CXCR7 activation promoted STAT3 signaling and vascular cell adhesion molecule-1 expression, accelerating disease progression (see Section 4) [131].

CXCL12-CXCR4 also shapes the tumor immune microenvironment. CXCL12 binds TNBC cells, impairing T-cell motility and preventing their infiltration, creating an immune-excluded phenotype associated with poor response to checkpoint inhibitors [148,149]. Moreover, the axis recruits immunosuppressive cells, including MDSCs, other myeloid suppressors, and Tregs, all linked to poor prognosis [150,151]. MDSCs inhibit T and natural killer (NK) cells through secretion of arginase 1, IL-10, and TGF-β, and by inducing Tregs [152]. CXCR4 blockade, in combination with anti-PD-1 therapy, reversed immunosuppression and enhanced T-cell responses in TNBC models [153,154]. Similarly, the CXCL12-CXCR7 axis promotes macrophage recruitment via the colony-stimulating factor-1/colony-stimulating factor-1 receptor pathway, further enhancing tumorigenesis and invasiveness [155].

Multiple strategies to inhibit CXCL12-CXCR4 signaling are being investigated, including CXCR4 antagonists, mAbs, and small-molecule inhibitors. The CXCR4 antagonist AMD3100 (plerixafor, MOZOBIL) showed tolerability and increased immune infiltration in early solid tumor studies. Several clinical trials evaluate CXCL12-CXCR4 inhibition. In metastatic pancreatic cancer, the trials have been completed, although results remain unavailable. In TNBC, clinical trial NCT05103917 launched X4P-001 (a CXCR4 allosteric inhibitor) combined with toriplimab (anti-PD-1) (Table 1), though no updated results have been reported in over two years [153,154].

## 4. Angiogenesis

Angiogenesis is the biological process by which new blood vessels form from preexisting vasculature. This dynamic mechanism is a crucial physiological process for tissue development, regeneration, and repair and is tightly regulated by a coordinated network of growth factors and complex signaling interactions [156]. Among these factors, the VEGF family, which includes structurally related proteins such as VEGF-A, VEGF-B, VEGF-C, VEGF-D, and placental growth factor (PIGF), serves as a central regulator of angiogenesis. VEGF exerts its biological effects primarily through interactions with specific tyrosine kinase receptors (VEGFR-1, VEGFR-2, and VEGFR-3), which mediate endothelial cell proliferation, migration, and vascular permeability [157].

Dysregulated angiogenesis, characterized by either insufficient or excessive neovascularization, plays a pivotal role in the pathogenesis and progression of diverse pathological conditions, including autoimmune diseases, chronic inflammatory disorders, and cancer [158]. Tumors rely on angiogenesis to meet their increasing metabolic demands. During early growth, tumors remain avascular and dormant, sustained by diffusion from adjacent tissues. However, as tumor volume exceeds 1–2 mm^3^, oxygen and nutrient diffusion become insufficient, leading to hypoxia, acidosis, and elevated interstitial pressure [159,160]. This unfavorable microenvironment triggers the upregulation of pro-angiogenic growth factors and cytokines, most notably VEGF and related mediators, which stimulate both angiogenesis and lymphangiogenesis, facilitating further tumor expansion [161]. The resulting neovasculature is often disorganized and inefficient, leading to persistent hypoxia and reinforcing angiogenic signaling in a self-perpetuating cycle [160]. Robust evidence has linked angiogenesis to the progression of a wide range of solid tumors, including breast [162], colorectal [163], lung [164], ovarian [165], renal [166], liver [167], melanoma [168], and glioblastoma [169].

TNBC is a highly angiogenic subtype of BC, characterized by significantly higher microvascular density (MVD) compared to other subtypes [170,171]. Elevated MVD has been associated with larger tumor size, advanced clinical stage, disease progression, and lymph node metastasis [172,173]. Moreover, elevated MVD correlates with poorer prognosis in patients with TNBC, highlighting the critical role of angiogenesis in its progression [171]. Patients with TNBC exhibit elevated intratumoral VEGF levels, which are associated with reduced recurrence-free and overall survival, as well as faster progression from initial diagnosis to relapse and from relapse to death [172,174].

Pro-inflammatory cytokines within TME play a critical role in orchestrating angiogenesis. Secreted by both tumor and stromal cells, these cytokines activate intracellular signaling cascades upon binding to their receptors, leading to the upregulation of angiogenic mediators such as VEGF. Key cytokines, including IL-6, IL-8, TGF-β, IL-1β, interferon gamma (IFN-γ), and TNF-α, modulate endothelial cell proliferation, migration, and survival, thereby shaping the angiogenic landscape of TNBC.

### 4.1. IL-6 and IL-8

TNBC cells secrete elevated levels of the pro-inflammatory cytokines IL-6 and IL-8, which are critical drivers of tumor progression. These cytokines not only activate oncogenic signaling pathways that support tumor cell proliferation and survival but also play a central role in promoting angiogenesis. As potent pro-angiogenic mediators, IL-6 and IL-8 facilitate tumor–endothelial cell crosstalk, thereby enhancing neovascularization [22,175,176]. Both cytokines can stimulate dormant microvascular endothelial cells, inducing their transition to an angiogenic phenotype and promoting the formation of capillary-like tubular structures [177].

IL-6 primarily exerts its pro-angiogenic effects through activation of the JAK/STAT3 signaling pathway, leading to the upregulation of *VEGF* and *MMPs*, which together promote endothelial cell proliferation, migration, and extracellular matrix remodeling [18,178,179]. Inhibition of IL-6 has been shown to reduce angiogenesis and the migratory potential of TNBC cells [22,180,181].

IL-8 promotes endothelial cell motility by modulating F-actin cytoskeletal organization through the PI3K–Rho/Ras-related C3 botulinum toxin substrate 1 (Rac1) axis [182,183]. Furthermore, IL-8 enhances the expression and enzymatic activity of MMP-2 and MMP-9 in endothelial cells, supporting extracellular matrix degradation and vascular remodeling during angiogenesis [184]. Additionally, IL-8 upregulates VEGF expression through CXCR2 and NF-kβ signaling pathways [117,185]. Interestingly, tocilizumab, an IL-6 receptor antagonist, reduces IL-8 levels in TNBC cells and exerts potent antiangiogenic effects. This activity is associated with the downregulation of VEGF-A and hypoxia-inducible factor-1α expression both in vitro and in vivo, further supporting its therapeutic potential in impairing TNBC-driven angiogenesis [35].

### 4.2. IL-1β

IL-1β is a key pro-inflammatory cytokine implicated in the progression of TNBC, primarily by enhancing angiogenesis and tumor invasiveness (see Section 3). IL-1β expression, induced by B cells, activates NF-κB signaling in tumor cells, upregulating proangiogenic genes such as *VEGF*, *IL-8*, and *MMP2* [186,187]. Clinically, elevated IL-1β expression in TNBC correlates with more advanced tumor stage, shorter recurrence-free survival, and reduced overall survival outcomes [186]. Furthermore, IL-1β activates p38 MAPK and Jun N-terminal kinase pathways in endothelial cells, inducing VEGF and angiopoietin-1, which stabilize newly formed vessels [188,189].

### 4.3. TNF-α

Although TNF-α can play a dual role in cancer biology, capable of exerting both tumor-suppressive and tumor-promoting effects (see Section 3), in TNBC, TNF-α is primarily associated with tumor progression, invasiveness, and angiogenesis [190]. Through NF-κB activation, TNF-α enhances transcription of *VEGF*, *IL-8*, *MMP2*, and *MMP9* [191]. This cascade supports endothelial cell proliferation and migration, contributing to neovascularization and tumor expansion. The neutralization of TNF-α using anti-TNF-α nanobodies significantly enhanced the therapeutic efficacy of bevacizumab, a VEGF inhibitor, in murine models of TNBC that had developed resistance to this treatment. The combination therapy not only reduced angiogenesis but also suppressed tumor progression and EMT, highlighting the therapeutic potential of targeting TNF-α in refractory TNBC [192].

### 4.4. VEGF

The VEGF family consists of five related proteins: VEGF-A, VEGF-B, VEGF-C, VEGF-D, and PIGF [193]. Among these, VEGF-A is the most studied and is the key regulator of blood vessel formation and permeability in both normal and disease states [157]. It exists in several isoforms. VEGF-B is mainly linked to cancer metastasis [194], while VEGF-C and VEGF-D are primarily involved in lymphangiogenesis [195], contributing to fluid regulation and immune function in various cancers. Beyond its proangiogenic role, VEGF also fosters an immunosuppressive TME by modulating immune cell recruitment and function [196,197]. Therefore, targeting VEGF signaling may not only inhibit angiogenesis but also restore anti-tumor immune responses, offering a promising therapeutic strategy for cancer treatment.

Clinically, several agents targeting the VEGF/VEGFR signaling pathway are currently utilized in the treatment of various cancers; however, none have received regulatory approval specifically for TNBC. Nevertheless, a substantial number of clinical trials have investigated the efficacy of mAbs and tyrosine kinase inhibitors targeting VEGF and its receptors in this subtype as monotherapy or in combination with chemotherapy and other targeted therapeutic agents (Table 2).

Bevacizumab (Avastin) was the first anti-angiogenic agent approved for clinical use. This humanized mAb binds all soluble isoforms of VEGF-A in circulation, effectively neutralizing their angiogenic activity [198]. In the Phase III RIBBON-2 trial (NCT00281697), a subgroup of 159 women with mTNBC treated with a combination of bevacizumab and a taxane showed significantly improved outcomes compared to chemotherapy alone: median progression-free survival (PFS) of 6.0 months vs. 2.7 months, overall survival (OS) of 17.9 vs. 12.6 months, and an objective response rate (ORR) of 41% vs. 18% [199].

In a Phase II study (NCT00691379), the combination of weekly paclitaxel, carboplatin, and bevacizumab in the first-line treatment for mTNBC achieved a median PFS of 10.3 months, OS of 25.7 months, and a duration of response of 18.2 months [200]. A subsequent trial (NCT03577743), in which patients were treated with bevacizumab, carboplatin, and paclitaxel every 21 days for six cycles, reported even more notable results, with a median PFS of 27 months and OS of 55 months [201]. These findings are consistent with data from other studies (NCT00479674, NCT00618657, NCT01094184, and NCT00733408), all of which demonstrated improved clinical outcomes, such as extended PFS and OS and higher partial response rates, when bevacizumab was combined with SOC chemotherapy.

However, not all trials have shown a survival benefit. The BEATRICE Phase III trial (NCT00528567), which enrolled approximately 2500 women with operable early-stage TNBC, failed to demonstrate a statistically significant OS benefit from adding bevacizumab to standard adjuvant chemotherapy [202]. Similarly, in the CALGB 40603 Phase II trial (NCT00861705), adding carboplatin or bevacizumab to weekly paclitaxel followed by doxorubicin and cyclophosphamide significantly increased pathological complete response (pCR) rates in Stages II–III TNBC but did not improve long-term survival outcomes [203]. Numerous ongoing or actively recruiting trials continue to explore bevacizumab-based combinations and dosing strategies to better define its therapeutic role in TNBC.

Apatinib is an orally administered small-molecule VEGFR-2 tyrosine kinase inhibitor currently under clinical investigation for TNBC. In a Phase II study (NCT03394287) [204], apatinib was combined with camrelizumab, a humanized anti-PD-1 antibody, based on the rationale that anti-angiogenic therapy could enhance tumor immune responsiveness by modulating the tumor microenvironment and normalizing vasculature, thereby improving immune cell infiltration and sensitivity to immunotherapy. This chemo-free combination achieved a notable ORR of 43.3%, surpassing previously reported outcomes for monotherapies with either anti-PD-1/PD-L1 antibodies or apatinib alone (NCT01176669) [205]. The regimen demonstrated encouraging anti-tumor activity with an acceptable safety profile in patients with advanced TNBC.

Building on this, the addition of the poly (ADP-ribose) polymerase (PARP) inhibitor fuzuloparib to the camrelizumab–apatinib combination was evaluated (NCT03945604) [206]. Although the safety profile remained manageable, the ORR was lower than in the prior study, potentially due to the higher apatinib dose used. Despite this, the disease control rate (DCR) and PFS were encouraging, warranting further exploration of this triplet therapy.

A related multicenter Phase II trial combined camrelizumab, apatinib, and eribulin, a chemotherapeutic agent (NCT04303741) [207]. This triplet achieved promising efficacy with an ORR of 37.0%, a DCR of 87.0%, and a median PFS of 8.1 months, while maintaining a manageable toxicity profile. In early, treatment-naïve TNBC, apatinib has also been investigated along with standard chemotherapy regimens. One study combining apatinib with docetaxel followed by epirubicin plus cyclophosphamide reported excellent clinical outcomes, including an ORR of 93.5%, a 24-month PFS of 90.9%, and a 24-month OS of 94.4%, with tolerable toxicity (NCT03243838) [208]. Another study comparing apatinib plus dose-dense paclitaxel and carboplatin against chemotherapy alone showed superior efficacy with acceptable adverse events, suggesting that apatinib may enhance outcomes without compromising safety (NCT03735082) [209].

Lenvatinib, a multi-target receptor tyrosine kinase inhibitor acting on VEGFR1–3, FGFR1–4, PDGFRα, RET, and c-KIT, has also shown promise in TNBC. The Phase II LEAP-005 trial (NCT03797326) [210] evaluated lenvatinib plus pembrolizumab in previously treated advanced TNBC and other solid tumors. This combination achieved a durable ORR of 32%, a manageable safety profile, and a median OS of 11.4 months as a second- or third-line treatment. Currently, follow-up clinical trials are actively recruiting patients to assess this combination in previously untreated TNBC (NCT04427293). Additional ongoing studies are testing lenvatinib combined with camrelizumab plus nab-paclitaxel in advanced first-line TNBC (NCT06849492) and with sindilimab plus nab-paclitaxel in recurrent or mTNBC (NCT06140576).

Fruquintinib is a selective oral tyrosine kinase inhibitor targeting VEGF receptors 1, 2, and 3 with high potency. A Phase I/1b trial (NCT03251378) assessing its safety and tolerability in mTNBC patients demonstrated preliminary anti-tumor activity, though further studies are necessary to confirm clinical efficacy. The ongoing trial NCT04577963 is evaluating fruquintinib in combination with tislelizumab, an anti-PD-1 antibody, in both treatment-naïve and previously treated advanced or mTNBC; however, results are pending.

Sunitinib, a VEGF and platelet-derived growth factor receptor kinase inhibitor, was assessed in a Phase II trial comparing sunitinib monotherapy to SOC chemotherapy in previously treated advanced TNBC. This study found no improvement in efficacy with sunitinib vs. SOC chemotherapy (NCT00246571) [211].

### 4.5. Implications of Anti-Angiogenic Approaches for Clinical Practice

New evidence underscores the potential of VEGF/VEGFR-targeted therapies to modulate the immunosuppressive tumor microenvironment in TNBC, thereby enhancing responsiveness to immune checkpoint inhibitors. Apatinib’s combinations with camrelizumab, chemotherapy, and PARP inhibitors show encouraging anti-tumor activity and manageable safety profiles, positioning these regimens as promising candidates for further development in both early and advanced settings. Similarly, lenvatinib’s multi-target profile and synergy with immunotherapy suggest it may overcome resistance mechanisms in heavily pretreated TNBC patients [206].

Future research should focus on optimizing dosing strategies to maximize efficacy while minimizing toxicity, understanding biomarkers predictive of response to VEGF-targeted immunotherapy combinations, and elucidating the mechanisms by which VEGF inhibition remodels the tumor immune milieu. Additionally, integration of these agents into earlier lines of therapy and combination with other targeted or immune-modulating treatments warrants further investigation to improve long-term outcomes in this aggressive BC subtype [212].

## 5. Cytokine-Mediated Modulation of TME and Immune Landscape

The TME comprises a complex and dynamic niche where malignant cells interact with non-malignant components, including stromal elements, infiltrating immune populations, vasculature, and the extracellular matrix. Key cellular and molecular constituents of the TME include CAFs, TAMs, TILs, MDSCs, and a diverse array of soluble mediators such as cytokines and chemokines. The TME plays a pivotal role in shaping immune evasion, therapeutic resistance, and clinical outcomes in TNBC by altering immune cell function and orchestrating cytokine secretion. Importantly, these cytokines not only modulate the immune landscape but also represent potential therapeutic targets for modulating the TME and improving treatment efficacy [213].

### 5.1. TAMs

TAMs are a predominant immune cell population within the TME of BC, particularly TNBC, where they contribute critically to establishing an immunosuppressive niche that supports tumor progression. Upon recruitment to the TME, macrophages polarize toward either classically activated (M1) or alternatively activated (M2) phenotypes. TAMs predominantly exhibit an M2-like phenotype characterized by the secretion of immunosuppressive cytokines, chemokines, and growth factors that drive pro-tumorigenic immune evasion, EMT, invasion, and metastasis. Key cytokines, including IL-10 and TGF-β, sustain this M2-like state by suppressing cytotoxic immune responses. Chemokines such as CCL2 and CCL5 facilitate monocyte recruitment and differentiation into TAMs, reinforcing a pro-tumorigenic feedback loop. Although TNF-α is classically pro-inflammatory, it can also support tumor cell survival and chronic inflammation, further promoting cancer progression. While the role of TAMs in tumor development is well established, the molecular mechanisms by which they regulate EMT and CSC induction require further elucidation [214].

Macrophages, as professional phagocytes and antigen-presenting cells (APCs) are critical for bridging innate and adaptive immunity [214,215]. In the TME, TAMs shift from a pro-inflammatory, tumoricidal M1 phenotype to an anti-inflammatory, tissue-repair-oriented M2 phenotype, thereby impairing the anti-tumor immune response [216,217,218]. This polarization reduces antigen recognition and phagocytic capacity and diminishes CD4^+^ and CD8^+^ T cell cytotoxicity. Furthermore, M2-like TAMs promote activation of Tregs and certain helper T cell subsets, fostering an immunosuppressive, pro-tumorigenic milieu [213,214,219].

#### 5.1.1. IL-1β

In TNBC, IL-1β promotes TAM recruitment, immunosuppression, and tumor progression, highlighting its relevance as a therapeutic target [220,221]. TNBC cells polarize macrophages toward a mixed M1/M2 phenotype, with TAMs exposed to TNBC-conditioned media expressing significantly higher levels of IL-1β than those exposed to hormone receptor-positive cancer cells [222]. Elevated IL-1β expression in TNBC patient tissues is paradoxically associated with delayed disease progression, suggesting a context-dependent or protective role in certain settings [222]. M2-like TAMs are key sources of IL-1β via inflammasome (particularly NLRP3) activation, supporting tumor progression through enhanced angiogenesis, immune evasion, and chronic inflammation [223]. IL-1β^+^ TAMs also modulate immune cell recruitment and polarization, reinforcing their role as pro-tumorigenic drivers and therapeutic targets [224].

IL-1β upregulates fibronectin containing the extra domain A (FN-EDA), a matrix component that in turn stimulates IL-1β production and activates NF-κB signaling in monocytes, creating a positive feedback loop that sustains the pro-inflammatory TME. This loop enhances monocyte-to-macrophage differentiation and TAM polarization toward a pro-tumorigenic phenotype [225].

In the ongoing clinical trials (Table 1), IL-1β inhibition is being assessed in BC, with special focus on analyzing immune biomarkers including TILs, TAMs, NK cells, IL-1β, and inflammasome components (see Section 3).

#### 5.1.2. IL-6

Beyond the various roles discussed in previous sections, IL-6 also promotes TAM polarization toward a pro-tumorigenic M2 phenotype and enhances secretion of cytokines and growth factors that support tumor invasion and metastasis. Through STAT3 activation, IL-6 promotes recruitment of Tregs and MDSCs, contributing to an immunosuppressive, pro-tumorigenic TME. IL-6 is a key regulator of inflammation [226], essential for host defense and cellular growth [227]. IL-6 pathways that influence TME by reducing fibroblast cell motility have also been described [228]. Several clinical trials are currently underway to evaluate the therapeutic potential of blocking IL-6 signaling (Table 1, see Section 2).

#### 5.1.3. CCL2

In recent years, strategies targeting TAMs have focused mainly on inhibiting their recruitment, depleting existing TAMs, or reversing their polarization. One key approach to block TAM recruitment is by targeting chemokines, particularly the CCL2/CCR2 axis, which reduces bone marrow monocyte mobilization and macrophage infiltration into breast tissue. Studies have shown that trabectedin and bortezomib can inhibit macrophage recruitment by lowering plasma CCL2 levels [229].

Bortezomib, a proteasome inhibitor primarily used to treat multiple myeloma, has been identified as a modulator of TAM polarization and the TME. It influences the expression of Krüppel-like factor 4 (KLF4), a transcription factor involved in alternative (M2) macrophage activation. By modulating KLF4 levels, bortezomib may shift TAMs from a pro-tumoral M2 phenotype to an anti-tumoral M1 phenotype, thereby enhancing the immune system’s ability to combat tumors [230]. Bortezomib also interferes with cytokine regulation by inhibiting the proteasome, leading to the accumulation of inhibitory proteins and suppression of NF-κB signaling, a key pathway that drives transcription of pro-inflammatory cytokines like CCL2. As a result, bortezomib reduces CCL2 production, leading to decreased monocyte recruitment and TAM differentiation, contributing to a less immunosuppressive microenvironment [229,231].

The phase I pilot study, NCT04265872, is currently investigating a combination therapy in women with mTNBC or ER-low BC (Table 3). The trial evaluates the safety, tolerability, and preliminary efficacy of administering bortezomib followed by pembrolizumab and cisplatin, aiming to sensitize cancer cells to cisplatin and enhance immune-mediated tumor elimination through pembrolizumab.

#### 5.1.4. CCL5

CCL5 (RANTES) plays a pivotal role in TNBC by recruiting TAMs via CCR1, CCR3, and CCR5. Once recruited, CCL5 induces TAM polarization toward a pro-tumoral M2 phenotype that promotes tumor progression, immunosuppression, angiogenesis, and metastasis [232]. Elevated CCL5 levels correlate with greater tumor invasiveness and immune evasion. Beyond macrophage recruitment, tumor-derived CCL5 also uses extracellular vesicles (EVs) to modify macrophage function, upregulating genes like *OPN*, *SLPI*, *HGF*, and *NRG3*, which promote invasion and metastasis [233].

Leronlimab, a humanized mAb targeting CCR5, has shown promising results in mTNBC across pooled analyses of three clinical trials (NCT03838367, NCT04313075, and NCT04504942) (Table 3). Administered as monotherapy or combined with chemotherapy, higher doses (≥525 mg weekly) were linked to a median progression-free survival of 6.1 months and overall survival beyond 12 months, with minimal grade 3 adverse events. Leronlimab reduced circulating tumor-associated cells and modulated CCR5-related cytokine production (CCL5, IL-6, and TNF-α), highlighting the potential of CCR5 blockade to reprogram the immunosuppressive microenvironment [234].

#### 5.1.5. TNF-α

TNF-α is one of the most important inflammatory cytokines among the numerous cytokines secreted in the TME [235,236]. TNF-α, which can exert both pro-apoptotic and pro-tumorigenic effects depending on the cellular context [84,85,237], also plays an important role in immune cell infiltration [11]. Elevated TNF-α levels are associated with proliferation, EMT, relapse, and metastasis in TNBC [87]. Cancer cells escape TNF-α-induced death by releasing soluble TNF receptors (sTNFR1/2), which neutralize TNF-α and promote tumor survival via reverse signaling. To enhance anti-tumor immunity and restore TNF-α activity, an innovative technology, Immunopheresis™, is being tested in a Phase I/II trial (NCT04004910) (Table 3). Immunopheresis™ is a novel extracorporeal therapy that removes sTNFR1/2 using a scTNF-functionalized column (LW-02). Early preclinical data and FDA Breakthrough Device Designation support this approach [238].

#### 5.1.6. TGF-β

TGF-β is a multifunctional cytokine regulating growth, differentiation, and immune responses [239]. In tumors, it promotes TAM immunosuppressive activity, M1-to-M2 polarization, and PD-L1 upregulation [239,240]. Additionally, TGF-β acts as a chemoattractant for monocytes. BC cells secrete TGF-β, increasing recruitment of CXCR4^+^ monocytes to perivascular fibroblasts producing CXCL12, forming a chemotactic gradient that promotes TAM migration toward the vasculature and facilitates tumor cell intravasation and dissemination [241].

#### 5.1.7. IFN-α

Cytokines play a dual role in cancer immunotherapy, functioning both as targets for inhibition and as therapeutic agents that can actively enhance anti-tumor immunity [242]. This is exemplified by interferon-alpha (IFN-α), a type I interferon with immunostimulatory and antiproliferative properties. In TNBC, type I interferons (including IFN-α and IFN-β) have been associated with favorable outcomes [242]. Specifically, higher expression of IFN-β in clinical TNBC samples correlates with suppression of CSC-related genes, increased infiltration of TILs, and ultimately improved patient survival [243,244].

Type I IFNs promote the differentiation of monocytes into dendritic cell-like APCs and drive precursor macrophages toward an anti-tumor M1 phenotype, both of which are crucial for initiating effective anti-tumor immune responses and enhancing tumor cell elimination. To exploit these properties therapeutically, a Phase I/IIa clinical trial (NCT05756166) is evaluating the combination of rintatolimod (a Toll-like receptor 3 agonist that stimulates endogenous type I IFN production), celecoxib (a COX-2 inhibitor that reduces immunosuppressive prostaglandins), interferon alpha-2b (directly boosting immune activation), and pembrolizumab (a PD-1 inhibitor that restores T cell function) (Table 3). This multi-agent approach aims to amplify innate and adaptive immune responses, enhance immune infiltration, and improve tumor control in patients with metastatic or unresectable TNBC. The trial is actively recruiting at Roswell Park Comprehensive Cancer Center and seeks to establish safety, optimal dosing, and preliminary efficacy of this combination.

### 5.2. TILs in TNBC: The Janus-Face of Anti-Tumor and Pro-Tumor Forces

A significant feature of the TME in TNBC is the proportion and type of TILs. TILs play a key role in modulating the immune escape mechanisms of tumors. The influence of TILs on progression and treatment response varies among BC subtypes. In TNBC, a high infiltration of TILs is associated with a better prognosis and a higher rate of pCR following neoadjuvant chemotherapy [245,246,247,248]. Even among patients who fail to achieve pCR after neoadjuvant chemotherapy, the presence of TILs in residual TNBC disease has been correlated with more favorable outcomes [249].

TILs, mainly composed of CD8^+^ T cells, CD4^+^ T cells, Tregs, NK cells, and B cells, orchestrate complex immune responses through the secretion of cytokines that can either promote anti-tumor immunity or support tumor progression [250]. Pro-inflammatory cytokines, such as IFN-γ, TNF-α, and IL-2, primarily secreted by CD8^+^ cytotoxic T cells and NK cells, are pivotal in mediating tumor-immune defense. In contrast, immunosuppressive cytokines, including IL-10 and TGF-β, facilitate immune evasion and may enhance metastatic potential.

Elucidating the cytokine landscape shaped by TILs in TNBC not only deepens our understanding of tumor immunology but also opens new avenues for clinical applications, such as cytokine-targeted therapies and cytokine-based immunotherapies.

#### 5.2.1. IL-2

IL-2 plays a key role in activating NK and T cells, essential for tumor immune surveillance and cytotoxicity. Its immunomodulatory properties make it a promising target in cancer immunotherapy. Recombinant IL-2 was one of the first cytokines approved by the FDA for metastatic melanoma and renal cell carcinoma, marking a milestone in the field [251,252].

Despite its therapeutic potential, demonstrated in numerous clinical trials [253], widespread clinical use has been limited by IL-2’s short half-life and substantial dose-limiting toxicities. Furthermore, native IL-2 preferentially expands immunosuppressive Tregs due to its high-affinity binding to IL-2 receptor α (IL-2Rαβγc or CD25/CD122/CD132), which is constitutively expressed on Tregs. This expansion can dampen anti-tumor responses and promote immune evasion within the TME.

In TNBC, IL-2-based therapies are being refined to enhance efficacy while minimizing systemic toxicity. Current approaches involve engineered IL-2 variants combined with immune checkpoint inhibitors (ICIs), mAbs, or adoptive T cell therapies to boost anti-tumor immunity. One such agent, bempegaldesleukin (NKTR-214/BEMPEG), is a PEGylated IL-2 receptor βγ-biased agonist that selectively stimulates CD122-IL-2 pathway signaling, thereby increasing TILs, T cell clonality, and PD-1 expression without promoting the unwanted expansion of Tregs in the TME of advanced solid tumors [254].

The clinical activity of BEMPEG was assessed in the Phase I PIVOT-02 trial (NCT02983045) (Table 3), where it was administered with nivolumab in patients with mTNBC [255]. Interestingly, clinical response may be associated with the presence of killer immunoglobulin-like receptor (KIR) ligands in TNBC, potentially enhancing NK cell anti-tumor activity [256]. Another approach, evaluated in the Phase Ib REVEAL trial (NCT03435640), combined BEMPEG with a toll-like receptor 7/8 agonist (NKTR-262) in patients with metastatic solid tumors, including TNBC (Table 3). This regimen aimed to activate local innate immune responses via TLR stimulation while synergizing with IL-2-driven adaptive immunity [257]. All BEMPEG-based clinical trials have been discontinued despite promising immunological effects, suggesting that IL-2 may lack efficacy as a monotherapy in TNBC and that its therapeutic potential likely depends on optimized combination strategies to improve outcomes.

#### 5.2.2. IL-12

IL-12 is a potent pro-inflammatory cytokine produced primarily by activated APCs, including dendritic cells, macrophages, monocytes, and B cells [258]. IL-12 initiates adaptive and anti-tumor immune responses by promoting the proliferation and activation of NK and T cells. It induces key effector molecules such as cytokines, perforin, and granzyme B [258,259].

IL-12 plays a crucial role in driving T helper 1 (Th1) differentiation and enhancing Th1 cell activity. A key factor in IL-12’s anti-tumor response is IFN-γ, which is secreted upon IL-12 stimulation of Th1, NK cells, and cytotoxic T lymphocytes (CTLs). IFN-γ also contributes to IL-12 secretion by APCs in a positive feedback loop [239]. Moreover, IL-12 helps maintain the Th1/Th2 balance and inhibits Treg and Th17 differentiation mediated by TGF-β [260]. IL-12 also regulates NK cell function, enhancing their cytotoxic activity and tumor cell destruction. It synergizes with T cell receptor signaling to induce effector CTL differentiation and contributes to their infiltration into the TME [261].

Although preclinical studies highlight the anti-tumor and anti-metastatic potential of recombinant IL-12 in various cancer models, including BC [262], severe immune-related adverse events have limited its clinical use. To overcome this, delivery methods under study include viral/non-viral vectors, chemical, and bio-based systems [263,264].

In TNBC models, G47Δ-mIL12, an engineered oncolytic virus expressing IL-12, suppressed tumor growth and prevented metastasis by enhancing APC activation, increasing CD8^+^ T cell infiltration, and reducing MDSCs [265]. Intratumoral injection of IL-12 plasmid (tavokinogene telseplasmid, tavo) followed by electroporation (IT-tavo-EP) drives local IL-12 exposure with minimal systemic toxicity. Preclinical studies in TNBC demonstrated that tavo therapy expanded and activated CD8^+^ T cells via a CXCR3 gene signature, induced PD-1/PD-L1 expression, and led to tumor regression and long-term survival [266]. The OMS-I140 trial (NCT02531425) confirmed these findings in advanced TNBC patients [266] (Table 3). The Phase II KEYNOTE-890 trial (NCT03567720) combined IT-tavo-EP with pembrolizumab in mTNBC, with preliminary results showing IL-12-driven immunologic responses, including MDSC reduction and increased sensitivity to pembrolizumab [267] (Table 3).

Chemo-gene co-delivery systems, such as HA/pIL-12/DOX-PMET nanoparticles, have shown synergistic chemo-immunotherapeutic effects in TNBC models by enhancing NK and TIL activity, polarizing M2 macrophages, reducing Tregs, and inhibiting metastasis [268]. IL-12 also synergizes with trabectedin to deplete MDSCs and M2-like TAMs, boost NK cell-mediated chemokine release, increase CD8^+^ T cell infiltration, and sensitize tumors to PD-L1 blockade [269].

Overall, IL-12 is a potent inducer of IFN-γ secretion that can promote Th1 immunity. Initial clinical trials with systemic IL-12 administration revealed significant toxicity and limited efficacy. To address these limitations, current strategies focus on enhancing safety and therapeutic potential through local delivery, tumor-targeted fusion proteins, and viral vectors [266].

#### 5.2.3. IFN-γ

IFN-γ is the only type II interferon, being a key pro-inflammatory cytokine. IFN-γ is predominantly produced by activated CD8^+^ CTLs, CD4^+^ Th1 cells, and NK cells within the TME. As a cytotoxic cytokine, IFN-γ induces tumor cell apoptosis, suppresses Treg function, promotes Th1 differentiation, and drives macrophage polarization toward an anti-tumor M1 phenotype, thereby enhancing anti-tumor immunity [270]. It promotes immune cell recruitment by inducing chemokines like CXCL9 and CXCL10, which attract effector T cells to the tumor and enhance local immune responses [242,270].

Clinically, IFN-γ contributes to the success of cancer immunotherapy and is increasingly recognized as a potential prognostic biomarker. In TNBC, high IFN-γ levels have been associated with increased TILs and improved clinical outcomes. IFN-γ also upregulates PD-L1 expression on tumor and immune cells, serving as a mechanistic predictor of response to ICIs, particularly anti-PD-1/PD-L1 therapies [271]. The BELLINI trial, which explored neoadjuvant ICIs without chemotherapy, reported higher baseline IFN-γ expression in patients who achieved pCR [272]. Similarly, the GeparNuevo trial demonstrated a strong correlation between TIL density, IFN-γ signatures, and pCR across the cohort [273].

Recent studies in aged murine models and TNBC patients have revealed a “cold” immune phenotype characterized by poor T cell infiltration and reduced IFN-related pathway expression, a determinant of ICI response. Remarkably, STING agonist-mediated stimulation of IFN signaling restored sensitivity to ICIs and improved survival in aged mice [274]. IFN-γ may further enhance anti-PD-L1 therapy by priming the immune system and increasing antigen presentation. A Phase I dose-escalation trial evaluating IFN-γ in combination with nivolumab in advanced solid tumors reported a complete response in one patient with mTNBC (NCT02614456) [275] (Table 3).

Importantly, MYC, frequently overexpressed in TNBC, promotes immune evasion by suppressing IFN-γ signaling [276]. IFN-γ, in turn, downregulates MYC expression, thereby enhancing major histocompatibility complex-I expression, CD8^+^ T cell infiltration, and responsiveness to anti-PD-L1 therapy, particularly in YC-driven tumors [277].

Despite its established anti-tumor properties, IFN-γ can also participate in regulatory feedback mechanisms that limit immune activation and promote tumor immune escape. Low-dose IFN-γ has been implicated in pro-tumorigenic activities. Singh et al. reported that the loss of the tumor-suppressive transcription factor Elf5 in TNBC led to IFN-γ pathway activation and accelerated tumor progression [278].

Collectively, IFN-γ plays a dual and context-dependent role in TNBC, acting as both an anti-tumor immune mediator and, at times, a facilitator of immune escape. Its capacity to recruit immune cells, boost antigen presentation, and synergize with ICIs makes it a promising therapeutic target and biomarker. However, its complex effects and toxicities require optimized delivery and combination strategies for effective treatment.

#### 5.2.4. IL-10

IL-10 is a pleiotropic cytokine that exerts both tumor-promoting and suppressive functions and plays a critical role in immune homeostasis. The role of IL-10 in BC remains controversial. Elevated serum IL-10 levels have been detected in BC patients compared to healthy controls; although IL-10 is a poor prognostic factor in several cancer types, it has been correlated with favorable survival outcomes in early-stage BC [279,280,281]. In TNBC, however, IL-10 is more consistently linked to poor prognosis. Toney et al. demonstrated that IL-10–producing tumor-infiltrating B cells promoted IgG4 isotype switching, a marker of poor prognosis in TNBC [282].

IL-10 mediates immunosuppression by inhibiting T cell proliferation, modulating antigen-presenting cell function, and sustaining Treg activity. In contrast, IL-10 has shown anti-tumor properties in specific contexts. Preclinical studies indicate that IL-10 can inhibit angiogenesis and MDA-MB-231 TNBC cell migration [283]. IL-10 also promotes activation of tumor-resident CD8^+^ T cells, retards tumor growth, and induces IFN-γ–dependent tumor cell death and metastasis suppression in murine BC models [284,285].

Given these contradictory roles, IL-10 remains a complex candidate for cancer immunotherapy. A major challenge is its short biological half-life and highly context-dependent immune activity. Pegilodecakin (AM0010), a PEGylated IL-10 formulation, was developed to overcome these limitations. Early clinical studies of pegilodecakin demonstrated Th1 immune stimulation and an acceptable safety profile in patients with advanced solid tumors (NCT02009449) (Table 3). When combined with anti-PD-1 therapies, pegilodecakin enhanced proliferation of PD-1^+^ Lag-3^+^ CD8^+^ T cells and expanded novel CD8^+^ T cell subsets [286]. A Phase Ib trial, including a unique patient with TNBC, evaluated pegilodecakin combined with nivolumab or pembrolizumab [287]. Moreover, pegilodecakin with docetaxel produced a synergistic 75% complete response in an ICI-resistant TNBC mouse model [288].

Although IL-10–based therapies exhibit anti-tumor potential and good tolerability, their clinical efficacy remains modest, underscoring the need for optimized delivery methods and combinatorial approaches in TNBC.

#### 5.2.5. TGF-β

TGF-β is a multifunctional cytokine abundantly expressed in the TNBC TME, where it contributes to immunosuppression by impairing TIL and DC activity, skewing macrophages toward a tumor-promoting phenotype, and inducing Treg differentiation [76]. TGF-β drives conversion of naïve CD4^+^ T cells into Tregs, reinforcing immune tolerance. It also impairs cytotoxic CD8^+^ T cell responses by suppressing DC-mediated antigen presentation and downregulating key cytokines such as IFN-γ and IL-2, thereby limiting CD8^+^ T cell proliferation [58].

Various anti-TGF-β strategies are under clinical and preclinical investigation (Table 1). Fresolimumab, an anti-TGF-β mAb, was evaluated in a Phase II trial for mBC patients receiving radiotherapy (NCT01401062). TGF-β blockade with fresolimumab enhanced systemic immunity, increased memory CD8^+^ T cells, and was associated with prolonged overall survival [289].

## 6. Future Perspectives

### 6.1. Bispecific Antibodies

In addition to traditional cytokine blockade strategies using mAbs or small-molecule inhibitors, bispecific antibodies (bsAbs) have emerged as a promising next-generation approach. BsAbs are engineered to simultaneously target two distinct molecules, offering the potential to modulate both cytokines and immune checkpoints within TME. One of the most advanced examples is bintrafusp alfa (M7824), a bifunctional fusion protein that combines PD-L1 blockade with a TGF-β “trap” via the extracellular domain of TGF-β receptor II. This dual targeting, also referred to as BiTP (bispecific anti-PD-L1/TGF-β protein) [290], has shown potent preclinical efficacy, suppressing both PD-L1–PD-1 and TGF-β–Smad signaling pathways. BiTP reduces collagen deposition, enhances CD8^+^ T cell infiltration, and increases overall TILs, leading to superior anti-tumor activity compared to single-agent therapies [291,292]. Based on promising preclinical and early-phase clinical data, bintrafusp alfa has been evaluated in multiple trials as monotherapy (NCT04489940) and in combination with other agents (NCT04296942, NCT03579472, and NCT04789668) (Table 4). The Phase I trial NCT04489940 enrolled TNBC patients with HMGA2 expression but was terminated early due to low probability of success [293]. Two Phase Ib trials, NCT04296942 (bintrafusp alfa + BN-brachyury vaccine) and NCT03579472 (bintrafusp alfa + eribulin), were also discontinued without posted results. The Phase I/II trial NCT04789668, which explored bintrafusp alfa with pimasertib in TNBC patients with brain metastases, has been completed; however, results are pending.

Bispecific strategies co-targeting VEGF and PD-L1 are also under clinical investigation (Table 4). BNT327 is in a Phase II trial (NCT06449222) for first- and second-line treatment of locally advanced or mTNBC in combination with chemotherapy. Similarly, B1962 is being evaluated in an ongoing trial (NCT06724263) for advanced solid tumors, including unresectable locally advanced or mTNBC.

### 6.2. Multi-Cytokine Targeting

Another innovative strategy is the use of multi-cytokine formulations to restore immune competence. IRX-2 is a cell-derived biologic composed of Th1-type cytokines (IL-2, IL-1β, IFN-γ, and TNF-α) produced by stimulated peripheral blood mononuclear cells (PBMCs). Preclinical studies show IRX-2 enhances dendritic cell function and T/NK cell activation, counteracting tumor-mediated immune evasion [294,295,296,297]. The neoIRX trial (Phase II, NCT04373031) is currently evaluating peri-lymphatic IRX-2 in combination with neoadjuvant pembrolizumab and chemotherapy for Stages II/III TNBC (Table 4). Preliminary findings indicate increased TILs, high pCR rates (83%), and good tolerability, supporting the promise of this approach [298].

### 6.3. Cytokine-Induced Killer Cells

Cytokine-induced killer (CIK) cell therapies, either alone or in combination with other strategies, represent promising anti-tumor approaches for BC patients, including those with TNBC. CIK cells are a heterogeneous population of ex vivo activated lymphocytes generated by stimulating PBMCs with cytokines such as IFN-γ, IL-1, IL-2, and anti-CD3 antibodies. As a result, CIK cells predominantly exhibit T cell and NK cell-like phenotypes and functions. They co-express CD3 and CD56 surface markers, and although a fraction of CIK cells express the T cell receptor complex, they display the cytotoxicity ability of T lymphocytes through a non-MHC-restricted [299,300]. The anti-tumor function of CIK cells is dependent on Fas–Fas ligand interaction and the release of perforin, granzyme, and numerous cytokines, such as IFN-γ and TNF-α [301].

Studies evaluating the clinical efficacy of CIK cell immunotherapy suggest that this therapy may serve as a valuable adjuvant strategy along with systemic chemotherapy in postoperative TNBC patients, particularly by reducing recurrence and metastasis rates. However, the long-term durability of these effects remains to be fully assessed [302]. Benefits appear more pronounced in patients diagnosed at earlier disease stages, as patients with advanced TNBC may exhibit impaired immune function, limiting the therapeutic potential of adoptive CIK transfer [303].

To explore the possibilities of CIK cells, their combinations have been extensively studied. Anti-tumor effects were found when CIK cells were combined with DCs in preclinical studies. The same strategy that has been widely implemented in clinical trials and has shown superior efficacy compared to conventional therapies [304]. Two observational studies evaluated this combinatory therapy in TNBC patients (NCT01395056, NCT01232062) [305] (Table 4). A similar strategy, combining adjuvant CIK therapy with NK cell-based immunotherapy, has shown promise in prolonging survival in BC patients post-mastectomy, with TNBC patients and those over 50 years of age deriving the greatest benefit, likely due to an enhanced cytotoxic immune environment [306].

Integrating CIK therapy with targeted agents has opened new therapeutic avenues. For example, combining CIK cells with cetuximab, an EGFR-specific chimeric IgG1 antibody, significantly inhibited tumor growth and metastasis in patient-derived TNBC xenograft models while increasing survival. This combinatory approach enhanced CD16a^+^ immune cell populations, contributing to antibody-dependent cellular cytotoxicity [307]. Similarly, FAK inhibitors sensitize TNBC cells to CIK-mediated killing via cGAS-STING pathway modulation and PD-L1 upregulation [308].

Despite these promising findings, CIK therapy is still rarely applied in clinical practice, mainly due to challenges associated with the heterogeneous and variable nature of CIK cells and their cytokine profiles. Nevertheless, the combination of CIK cell-based immunotherapy with other anti-tumor procedures holds considerable promise in cancer treatment, particularly as an alternative for elderly patients who are unable to tolerate surgery or chemotherapy [299].

### 6.4. TRUCKs: Activating Cytokines Secreted by CAR-T

Chimeric antigen receptor (CAR)-T cells are T lymphocytes engineered to express synthetic receptors targeting specific tumor-associated antigens. CAR-T therapy has achieved remarkable success in hematological malignancies, but its efficacy in solid tumors, such as TNBC, remains limited. Numerous preclinical efforts are focused on enhancing CAR-T efficacy by targeting immunosuppressive components of the TME and integrating combinatorial cytokine modulation to boost anti-tumor immunity [309]. The current landscape of CAR-T therapies for TNBC features innovative preclinical and early-phase clinical strategies to overcome TME barriers and enhance cell persistence. Fourth-generation CAR-T designs, known as TRUCKs (T cells Redirected for Universal Cytokine Killing), secrete cytokines such as IL-12, IL-15, IL-7, or IL-18 upon antigen engagement. This secretion recruits endogenous immune cells and amplifies anti-tumor responses [310,311]. In preclinical TNBC models, CAR-T cells co-expressing IL-15 or IL-7 exhibited improved proliferation, trafficking, and persistence, while TRUCKs delivering IL-12 recruited macrophages and mediated bystander killing of antigen-negative cells [312].

Several TRUCK designs are under investigation (Table 4). For instance, C7R-based CAR-T cells, which provide IL-7 signaling, are being explored in preclinical studies [312] and early-phase clinical trials (NCT04099797). However, CAR-T persistence remains suboptimal, and cytokine engineering is considered essential to improve efficacy [310,313,314,315]. Collectively, these findings highlight cytokine modulation as a key element in CAR-T manufacturing and design, offering a promising avenue for TNBC immunotherapy.

### 6.5. Next-Generation CAR-NK Cells

While CAR-T therapy has revolutionized hematologic cancer treatment, its use in solid tumors is limited by adverse events such as cytokine release syndrome. CAR-engineered NK cells, with their distinct cytokine secretion profiles and lack of clonal expansion, may help overcome these challenges. Their allogeneic compatibility, along with a reduced risk of graft-vs.-host disease, cytokine storm, or tumor lysis syndrome, positions CAR-NK cells as a promising alternative for solid tumor immunotherapy [316].

In TNBC, CD44v6, an adhesion molecule implicated in tumorigenesis and metastasis, has emerged as a target for next-generation CAR-NK therapy. Novel CAR-NK constructs targeting CD44v6 incorporate an IL-15 superagonist and a PD-1 checkpoint inhibitor. The IL-15 superagonist is released upon CD44v6 recognition, enhancing the cytotoxic response, while PD-1 blockade counters the immunosuppressive TME, which is characterized by upregulated PD-1 ligands in TNBC [317]. This multi-pronged CAR-NK strategy, designed to enhance targeting, cytotoxicity, and immune activation, offers a promising therapeutic option for BC, including TNBC.

## 7. Materials and Methods

This narrative review was conducted using a structured and transparent methodology. A focused PubMed search was performed using combinations of keywords such as ‘cytokines,’ ‘triple-negative breast cancer,’ and specific cytokine names. Studies were included if they focused on TNBC and provided mechanistic insights, therapeutic implications, or clinical relevance. Non-specific studies or those lacking experimental support were excluded. Relevant clinical trials were identified via ClinicalTrials.gov and PubMed. This strategy ensured a comprehensive coverage of the most relevant and current findings in the field.

## 8. Conclusions

Cytokines play a central role in TNBC, orchestrating tumor proliferation, invasion, metastasis, angiogenesis, and immune evasion. Targeting cytokine signaling offers a promising avenue for overcoming therapeutic resistance and improving patient outcomes, while also preparing TME to enhance the efficacy of immunotherapy and cellular therapies. Ongoing trials, including those exploring bispecific antibodies, are expected to refine these strategies and advance precision immunotherapy for TNBC.

## Figures and Tables

**Table 1:** Summary of clinical trials targeting the most important cytokines in proliferation and dissemination in TNBC.

Cytokine	Clinical Trial ID	Treatment	Phase Trial Stage	Status	Results	Disease Setting
IL-6	NCT05846789	SOC chemotherapy ± **Tocilizumab**	Phase II	Recruiting	No results posted	mTNBC or ER low BC
	NCT06008275	Neratinib + **Ruxolitinib**	Phase I	Recruiting	No results posted	mTNBC
	NCT02876302	**Ruxolitinib** ± Paclitaxel ± Doxorubicin ± Cyclophosphamide	Phase II	Active, not recruiting	1st R + 2nd P pCR: 83.3%; DFS: 66.7%; OS:50% R + P pCR: 94.1%; DFS: 53.3%; OS: 29.4%	TNBC
	NCT02041429	**Ruxolitinib** + Paclitaxel	Phase I/II	Completed	R + P: MTD: 25 mg bid RP2D: 15 mg bid 68% of patients required dose reductions; PR: 21%; SD: 63%; DP: 16%	HER2-negative mBC
IL-8	NCT02001974	Paclitaxel + **Reparixin**	Phase I	Completed	Group 1: P + R oral 400 mg tid; CBR: 25.0%; 6-mths PFS: 25%; TTP: 58 Group 2: P + R oral increase to 800 mg tid; CBR: 33.3%; 6-mths PFS: 0%; TTP: 67 Group 3: P + R increase to 1200 mg tid; CBR: 56.5%; 6-mths PFS: 17.4%; TTP: 170	HER2-negative mBC
	NCT02370238	Paclitaxel + **Reparixin**	Phase II	Completed	Weekly administration is safe and tolerable	mTNBC
TGF-β	NCT03012230	**Ruxolitinib** + Pembrolizumab	Phase I	Completed	No results posted	TNBC
	NCT02672475	**Galunisertib +** Paclitaxel	Phase I	Completed	No results posted	Metastatic AR-negative TNBC
	NCT02725489	**Vigil** + Durvalumab	Phase II	Completed	No results posted	Advanced/metastatic women’s cancers
	NCT01401062	**Fresolimumab** + radiotherapy	Phase II	Completed	Good immune response, increase in CD8 T cells, and longer median OS	mBC
	NCT02947165	**NIS793** + PDR001	Phase I/Ib	Completed	No results posted	Advanced/metastatic tumors, including TNBC
IL-1	NCT02900664	PDR001 in combination with CJM112, EGF816, **Canakinumab** or Trametinib	Phase Ib	Completed	No results posted	Advanced/metastatic cancer, including TNBC
	NCT01802970	**Anakinra** ± Nab-paclitaxel ± Capecitabine ± Eribulin ± Vinorelbine	Phase Ib	Completed	No results posted	HER2-negative mBC
	NCT03742349	Spartalizumab + LAG525 ± NIR178 ± Capmatinib ± MCS110 ± **Canakinumab**	Phase Ib	Terminated	No results posted	Advanced or mTNBC
	NCT06710197	**Anakinra**	Phase II	Recruiting	No results posted	Early-stage TNBC and ER-low BC
CXCL12	NCT05103917	**X4P-001** + Toriplimab	Phase I/II	Unknown status	No results posted	Locally advanced/mTNBC/

Abbreviations: triple negative breast cancer (TNBC); metastatic TNBC (mTNBC); breast cancer (BC); metastatic BC (mBC); estrogen receptor (ER); standard of care (SOC); ruxolitinib (R); paclitaxel (P); reparixin (R); maximum tolerated dose (MTD); recommended phase 2 dose (RP2D); partial response (PR); stable disease (SD); disease progression (DP); three times daily (tid); clinical benefit rate (CBR); progression-free survival (PFS); tumor progression in days (TTP); months (mths).

**Table 2:** Summary of clinical trials targeting VEGF in TNBC.

Clinical Trial ID	Treatment	Phase Trial Stage	Status	Results	Disease Setting
NCT00281697	**Bevacizumab** + Taxane vs. Taxane	Phase III	Completed	PFS 6 mths vs. 2.7 mths; OS 17.9 mths vs. 12.6 mths; ORR 41% vs. 18%	mTNBC
NCT00691379	**Bevacizumab** + Paclitaxel + Carboplatin	Phase I/II	Completed	ORR 65.2%; PFS 10.3 mths; OS 25.7 mths	mTNBC
NCT03577743	**Bevacizumab +** Carboplatin + Paclitaxel	Phase II	Completed	PFS 27 mths; OS 55 mths;	mTNBC
NCT00479674	**Bevacizumab** + Nab-paclitaxel + Carboplatin	Phase II	Completed	CR 18%; PR 69%; PFS 15 mths	mTNBC (Stage IV or inoperable Stage III)
NCT00618657	**Bevacizumab** + Carboplatin + Nab-paclitaxel	Phase II	Completed	PFS 93% 2 years; 17.6% pCR	Stages I–III TNBC
NCT01094184	**Bevacizumab** + Paclitaxel + Docetaxel	Phase IV	Completed	OS 11.6 mths (10 mg/kg) 18.9 mths (15 mg/kg); PFS 6.7 mths (10 mg/kg) 7.9 mths (15 mg/kg)	Advanced TNBC
NCT00733408	**Bevacizumab** + Nab-Paclitaxel + Erlotinib	Phase II	Completed	PFS 9.1 mths; OS 18.1 mths; PR 74%	mTNBC
NCT00528567	**Bevacizumab** + Chemotherapy vs. Chemotherapy	Phase III	Completed	IDFS: 31.5 mths vs. 21 mths; no difference OS	Operable primary invasive TNBC
NCT00861705	**Bevacizumab** ± Doxorubicin + Cyclophosphamide Paclitaxel ± Carboplatin	Phase II	Active	ORR 32%; PFS 5.1 mths; DR 12.1 mths; OS 11.4 mths	Previously treated TNBC
NCT03394287	**Apatinib** (daily or intermittent) + Camrelizumab	Phase II	Completed	ORR 43.3% (daily), PFS 3.7 mths vs. 1.9 mths; DCR 63.3% vs. 40%	Advanced TNBC
NCT01176669	**Apatinib**	Phase II	Completed	PFS 3.8 mths; OS 10.6 mths; OR 17.5%	Pretreated mTNBC
NCT03945604	**Apatinib** + Camrelizumab + Fuzuloparib	Phase Ib	Completed	ORR 6.9%; PFS 5.2 mths; OS 64.2% 12 mths	Recurrent and mTNBC
NCT04303741	**Apatinib** + Camrelizumab + Eribulin	Phase II	Completed	ORR 37%; PFS 8.1 mths;	Advanced TNBC
NCT03243838	**Apatinib** + Docetaxel + Epirubicin + Cyclophosphamide	Phase II	Completed	pCR 54.8%; OR: 93.5%; PFS 90.9% 24 mths; OS 94.4% 24 mths	Untreated stages IIA-IIIB TNBC
NCT03735082	**Apatinib** ± Paclitaxel ± Carboplatin	Phase II	Completed	ORR 88%; pCR: 60.9% vs. 30.4%	Locally advanced TNBC
NCT03797326	**Lenvatinib** + Pembrolizumab	Phase II	Completed	ORR 32%; PFS 5.1 mths; DR 12.1 mths; OS 11.4 mths	Advanced previously treated TNBC
NCT04427293	**Lenvatinib** + Pembrolizumab	Phase I	Recruiting	/	Untreated TNBC
NCT06849492	**Lenvatinib** + Camrelizumab + Nab-paclitaxel	Phase II	Active	/	Advanced first-Line TNBC
NCT06140576	**Lenvatinib** + Sindilimab + Nab-paclitaxel	Phase Ib/IIa	Active	/	Recurrent and mTNBC
NCT03251378	**Fruquintinib**	Phase I	Completed	PFS 38.46% 29 mths	mTNBC
NCT04577963	**Fruquintinib** + Tislelizumab	Phase Ib/2	Completed	No results posted	Advanced/mTNBC
NCT00246571	**Sunitinib** vs. chemotherapy (Capecitabine/Vinorelbine/Docetaxel/Paclitaxel/Gemcitabine)	Phase II	Completed	PFS 1.7 mths vs. 2.5 mths SOC; ORR 8.8 vs. 11.5%; OS 9.4 vs. 10.5 mths	Previously advanced treated TNBC

Abbreviations: triple negative breast cancer (TNBC); metastatic TNBC (mTNBC); progression-free survival (PFS); overall survival (OS); objective response rate (ORR); pathologic complete response (pCR); partial response (PR); complete response (CR); months (mths); standard of care (SOC); duration response (DR); disease control rate (DCR).

**Table 3:** Summary of clinical trials targeting the most important cytokines in immunomodulation in TNBC.

Cytokine	Clinical Trial ID	Treatment	Phase Trial Stage	Status	Results	Disease Setting
CCL2	NCT04265872	**Bortezomib** + Pembrolizumab + Cisplatin	Phase I	Recruiting	/	mTNBC
CCL5	NCT03838367	**Leronlimab** + Carboplatin	Phase Ib/II	Active, not recruiting	Results submitted, not reviewed	mTNBC
	NCT04313075	**Leronlimab** + TPC	Compassionate Use	No longer available	No results posted	mTNBC
	NCT04504942	**Leronlimab**	Phase II	Unknown status	No results posted	Locally advanced/mTNBC
TNF-α	NCT04004910	**Immunopheresis^®^ LW-02 column**	Phase I/II	Unknown status	No results posted	Advanced/refractory BC
IFN-α	NCT05756166	Rintatolimod, Celecoxib, and **IFN-α 2b +** Pembrolizumab	Phase I/IIa	Terminated	No results posted	mTNBC/unresectable TNBC
IFN-γ	NCT02614456	**Interferon-gamma** and Nivolumab	Phase I	Completed	No results posted	mTNBC
IL-2	NCT02983045	**Bempegal desleukin** + Nivolumab	PIVOT-02, Phase I	Terminated	ORR of 21% in the >2/3L metastatic patients, with an ORR of 23% in >2/3L metastatic patients with PD-L1 negative baseline. Patients with known pre-treatment PD-L1 status: ORR 14% in PD-L1 negative patients and 17% in PD-L1 positive patients. DCR 45%	Locally advanced/mTNBC
	NCT03435640	**Bempegal desleukin** + NKTR-262 TLR 7/8 agonist + Nivolumab	REVEAL, Phase Ib	Terminated	ORR 14.3% significant expansion of activated CD8^+^ T cells in BEMPEG + NKTR-262	Locally advanced/mTNBC
IL-12	NCT02531425	**IT-pIL12-EP**	OMS-I140, Phase I	Completed	No results posted	Locally advanced, inoperable, mTNBC/or treatment-refractory TNBC
	NCT03567720	**IT-pIL12-EP** + Pembrolizumab	KEYNOTE-890, Phase II	Unknown	ORR 17.4%, median DCR 16.6 mths, median OS 11 mths	Locally advanced/mTNBC
IL-10	NCT02009449	**Pegilodecakin** + Platinum and taxane-based chemotherapy1 or platinum and gemcitabine chemotherapy2	Phase I/Ib	Completed	ORR 28.6% plus chemotherapy1; DCR 25% monotherapy vs. 71.4% chemotherapy1 vs. 55.6 chemotherapy2; mPFS 2 mths vs. 3.9 mths chemotherapy1 vs. 3.7 mths chemotherapy2; mOS 5.3 mths vs. 11.2 mths chemotherapy1 vs. 6.8 mths chemotherapy2	Advanced solid tumors

Abbreviations: triple negative breast cancer (TNBC); metastatic TNBC (mTNBC); objective response rate (ORR); disease control rate (DCR); median progression-free survival (mPFS); median overall survival (mOS); months (mths).

**Table 4:** Summary of new strategies targeting cytokines in TNBC clinical trials.

Cytokine/s	Clinical Trial ID	Treatment	Phase Trial Stage	Status	Results	Disease Setting
TGF-β	NCT04489940	**Bintrafusp Alfa**	Phase II	Terminated	No results posted	mTNBC
	NCT04296942	**Bintrafusp Alfa** + BN-brachyury	BrEAsT, Phase Ib	Terminated	No results posted	Advanced BC
	NCT03579472	**Bintrafusp Alfa** + Eribulin mesylate	Phase I	Terminated	No results posted	mTNBC
	NCT04789668	**Bintrafusp Alfa** + pimasertib	Phase I/II	Completed	No results posted	Brain metastatic, including TNBC
VEGF	NCT06449222	**BNT327** + Nab-paclitaxel ± Carboplatin ± Gemcitabine ± Paclitaxel ± Eribulin	Phase II	Recruiting	/	Locally advanced/mTNBC
	NCT06724263	**B1962** Injection	Phase II	Active	/	Locally advanced/mTNBC
IL2, IL1β, IFN-γ, and TNF-α	NCT04373031	**IRX-2** + Pembrolizumab	neoIRX, Phase II	Active, not recruiting	pCR 83% vs. 33% pembro alone	Stages II/III TNBC
CIK	NCT01395056	**CIK** + Chemotherapy	Observational	Completed	Prevented disease recurrence and prolonged survival	mTNBC
	NCT01232062	**CIK +** High-Dose Chemotherapy	Observational	Completed	Time to disease progression 6 to 12 months; survival rates 1 year	mTNBC
TRUCK	NCT04099797	**C7R-GD2.CAR T cells** + Lymphodepletion	Phase I	Completed	PR 29%; SD 71%; cytokine release syndrome Grade 1–Grade 4 (75%); TIAN 88%; neurological improvement 90%	GD2-expressing brain tumors (GAIL-B)

Abbreviations: triple negative breast cancer (TNBC); metastatic TNBC (mTNBC); pathologic complete response (pCR); partial response (PR); stable disease (SD); tumor inflammation-associated neurotoxicity (TIAN).

## Data Availability

Data sharing not applicable.

## Abbreviations

The following abbreviations are used in this manuscript:

Akt	Protein Kinase B
APCs	Antigen-Presenting Cells
BC	Breast Cancer
Bcl-2	B-cell leukemia/lymphoma 2 protein
BCSCs	Breast Cancer Stem Cells
CAFs	Cancer-associated fibroblasts
CAR	Chimeric Antigen Receptor
CCLX	C-C motif chemokine ligand type X
CCRX	C-C chemokine receptor type X
CDX	Cluster of Differentiation type X
CIK	Cytokine-induced killer
CSCs	Cancer Stem Cells
CTLs	Cytotoxic T lymphocytes
CTX	Cetuximab
CXCL12	C-X-C motif chemokine ligand 12
CXCRX	C-X-C motif chemokine receptor type X
DC	Dendritic Cells
DCR	Disease Control Rate
DOX	Doxorubicin
DP	Disease Progression
DPP	Dipeptidyl Peptidase
EGFR	Epidermal Growth Factor Receptor
EMT	Epithelial-to-Mesenchymal Transition
EP	Electroporation
EVs	Extracellular vesicles
FAK	Focal Adhesion Kinase
FN-EDA	Fibronectin containing the Extra Domain A
HER2	Human Epidermal Growth Factor Receptor 2
IBC	Inflammatory BC
ICI	Immune checkpoint inhibitors
ICIs	Immune Checkpoint Inhibitors
IFN-γ	Interferon gamma
IL-X	Interleukin-type X
IrAEs	Immune-related Adverse Events
JAK	Janus Kinase
KIR	Killer immunoglobulin-like receptor
KLF4	Krüppel-like factor 4
mAb	monoclonal Antibody
MAPK	Mitogen-Activated Protein Kinase
mBC	Metastatic Breast Cancer
MCL-1	Myeloid Cell Leukemia-1
MDR	Multi-drug Resistance
MDSC	Myeloid-Derived Suppressor Cells
MET	Mesenchymal–Epithelial Transition
MMP9	Matrix Metalloproteinase-9
MMPs	Metalloproteinases
MTD	Maximum Tolerated Dose
MVD	Microvascular Density
mTNBC	Metastatic Triple Negative Breast Cancer
mTOR	Mammalian Target of Rapamycin
NACT	Neoadjuvant Chemotherapy
NF-κB	Factor Nuclear Kappa B
NK	Natural Killer
OS	Overall Survival
ORR	Objective Response Rate
OV	Oncolytic Virus
PARP	Poly (ADP-ribose) Polymerase
PBMCs	Peripheral Blood Mononuclear Cells
pCR	Pathologic Complete Response
PD-1	Programmed Death Protein 1
PD-L1	Programmed Death-Ligand 1
PFS	Progression-Free Survival
PIGF	Placental Growth Factor
PI3K	PhosphatidylInositol-3 Kinase
pIL12	Il-12 plasmid
PLC	Phospholipase C
PR	Partial disease Responses
PTEN	Phosphatase and Tensin homolog
Rac1	Ras-related C3 botulinum toxin substrate 1
Rho	Rhodopsin
sIL-6R	Soluble form of IL-6R
SMADX	Suppressor of Mother Against Decapentaplegic Type X
SOC	Standard of Care
STAT	Signal Transducer and Activator of Transcription
STING	Stimulator of Interferon Genes
TAMs	Tumor-Associated Macrophages
TF	Transcription Factors
TGF-β	Transforming growth factor-beta
ThX	T-helper type X
TIBs	Tumor-Infiltrating B cells
TILs	Tumor Infiltrating Lymphocytes
TLR	Toll-like Receptor
TME	Tumor Microenvironment
TNBC	Triple Negative Breast Cancer
TNF-α	Tumor Necrosis Factor Alpha
Treg	Regulatory T cell
TRUCKs	T cells Redirected for Universal Cytokine Killing
VEGF	Vascular Endothelial Growth Factor
